# The Exosomal/Total α-Synuclein Ratio in Plasma Is Associated With Glucocerebrosidase Activity and Correlates With Measures of Disease Severity in PD Patients

**DOI:** 10.3389/fncel.2018.00125

**Published:** 2018-05-18

**Authors:** Silvia Cerri, Cristina Ghezzi, Maria Sampieri, Francesca Siani, Micol Avenali, Gianluca Dornini, Roberta Zangaglia, Brigida Minafra, Fabio Blandini

**Affiliations:** ^1^Laboratory of Functional Neurochemistry, IRCCS Mondino Foundation Pavia, Italy; ^2^Neurological Rehabilitation Unit, IRCCS Mondino Foundation Pavia, Italy; ^3^Department of Brain and Behavioral Sciences, University of Pavia Pavia, Italy; ^4^Immunohemeatology and Transfusion Service, Fondazione IRCCS Policlinico San Matteo Pavia, Italy; ^5^Parkinson’s Disease and Movement Disorders Unit, IRCCS Mondino Foundation Pavia, Italy

**Keywords:** α-synuclein, glucocerebrosidase, exosomes, biomarkers, Parkinson’s disease

## Abstract

Intensive research efforts in the field of Parkinson’s disease (PD) are focusing on identifying reliable biomarkers which possibly help physicians in predicting disease onset, diagnosis, and progression as well as evaluating the response to disease-modifying treatments. Given that abnormal alpha-synuclein (α-syn) accumulation is a primary component of PD pathology, this protein has attracted considerable interest as a potential biomarker for PD. Alpha-synuclein can be detected in several body fluids, including plasma, where it can be found as free form or in association with exosomes, small membranous vesicles secreted by virtually all cell types. Together with α-syn accumulation, lysosomal dysfunctions seem to play a central role in the pathogenesis of PD, given the crucial role of lysosomes in the α-syn degradation. In particular, heterozygous mutations in the GBA1 gene encoding lysosomal enzyme glucocerebrosidase (GCase) are currently considered as the most important risk factor for PD. Different studies have found that GCase deficiency leads to accumulation of α-syn; whereas at the same time, increased α-syn may inhibit GCase function, thus inducing a bidirectional pathogenic loop. In this study, we investigated whether changes in plasma total and exosome-associated α-syn could correlate with disease status and clinical parameters in PD and their relationship with GCase activity. We studied 39 PD patients (mean age: 65.2 ± 8.9; men: 25), without GBA1 mutations, and 33 age-matched controls (mean age: 61.9 ± 6.2; men: 15). Our results showed that exosomes from PD patients contain a greater amount of α-syn compared to healthy subjects (25.2 vs. 12.3 pg/mL, *p* < 0.001) whereas no differences were found in plasma total α-syn levels (15.7 vs. 14.8 ng/mL, *p* = 0.53). Moreover, we highlighted a significant increase of plasma exosomal α-syn/total α-syn ratio in PD patients (1.69 vs. 0.89, *p* < 0.001), which negatively correlates with disease severity (*p* = 0.014). Intriguingly, a significant inverse correlation between GCase activity and this ratio in PD subjects was found (*p* = 0.006). Additional and large-scale studies comparing GCase activity and pathological protein levels will be clearly needed to corroborate these data and determine whether the association between key players in the lysosomal system and α-syn can be used as diagnostic or prognostic biomarkers for PD.

## Introduction

Parkinson’s disease (PD) is a common neurodegenerative disorder characterized by the degeneration of dopaminergic neurons in the substantia nigra (SN). One of the major pathological characteristics of PD is the presence of Lewy bodies composed of fibrillar aggregations of misfolded alpha-synuclein (α-syn; Spillantini et al., 1997). In PD there is a large time-gap between the beginning of the neurodegenerative processes and the onset of clinical manifestations, since motor symptoms are developing when there is already approximately 60%–80% of striatal dopaminergic loss (Cooper and Chahine, 2016). Moreover, motor features of PD progress at variable rates in different individuals (Schapira and Schrag, 2011). Therefore, the identification of reliable PD-specific biomarkers able to recognize individuals “at risk” before motor symptoms appear, improve PD diagnosis and monitor disease progression is a critical unmet need in the field.

The development of biochemical markers is most logically based on the understanding of processes involved in disease pathogenesis and progression. Given that abnormal α-syn accumulation is a primary component of PD pathology, this protein has attracted considerable interest as a potential biomarker for PD (Simonsen et al., 2016). Alpha-syn containing inclusions are not restricted to the central nervous system, but can also be stained in peripheral body tissues confirming the systemic nature of α-syn pathology (Braak et al., 2003). Moreover, α-syn can be detected in several body fluids (Malek et al., 2014). Plasma and saliva are increasingly investigated as alternative source to assess α-syn (Al-Nimer et al., 2014; Shi et al., 2014; Vivacqua et al., 2016) being more easily accessible than cerebrospinal fluid (CSF). Moreover, conflicting results obtained in studies on α-syn species in CSF further stimulated biomarker discovery in these biological fluids (Malek et al., 2014).

It is now accepted that α-syn possesses the ability to self-propagate throughout the brain. One potential mechanism of α-syn spreading is through exosomes (Emmanouilidou et al., 2010; Danzer et al., 2012; Coleman and Hill, 2015)—small membranous vesicles secreted by virtually all cell types, including neurons (Lachenal et al., 2011)—that can easily be isolated from conditioned cell media or bodily fluids. The involvement of exosomes in transmitting α-syn pathology has been clearly demonstrated, *in vitro* and *in vivo* (Lee et al., 2005; Danzer et al., 2012). Moreover, growing evidence suggests that exosomes can provide more reliable biomarkers for neurodegenerative diseases than biological fluids *per se*, since they carry unique, disease-specific cargos reflecting changes occurring during the disease (Vella et al., 2016; Wu et al., 2017). Accordingly, exosomes harboring neurodegeneration-related proteins have been detected in blood, CSF, urine and saliva of patients with neurodegenerative diseases (Ho et al., 2014; Vella et al., 2016; Taymans et al., 2017). To the best of our knowledge, only one study has so far examined α-syn content in plasma exosomes from patients with PD, disclosing that levels of α-syn in brain (L1CAM-positive) derived-exosomes were substantially higher in PD patients than in control subjects (Shi et al., 2014).

Together with α-syn accumulation, lysosomal dysfunctions seem to play a central role in the pathogenesis of PD, given the crucial role of lysosomes in α-syn degradation. In particular, heterozygous mutations in the GBA1 gene encoding lysosomal enzyme glucocerebrosidase (GCase) are currently considered the most important risk factor for PD, as 5% to 10% of all PD patients carry these mutations (McNeill et al., 2012; Sidransky and Lopez, 2012). Homozygous mutations of GBA1 cause Gaucher’s disease (GD), a common lysosomal storage disorder. GCase is responsible for the conversion of glucocerebroside to glucose and ceramide. *In vitro* studies have found that GCase deficiency leads to accumulation of α-syn (Bae et al., 2015); at the same time, increased α-syn may inhibit GCase function, thus inducing a bidirectional pathogenic loop (Manning-Bog et al., 2009; Mazzulli et al., 2011; Schapira, 2015). Lysosomal enzymes have been explored as potential diagnostic biomarkers in CSF, suggesting their activity could provide a biochemical fingerprint of PD, particularly if combined with the evaluation of pathological proteins (van Dijk et al., 2013; Parnetti et al., 2014; Moors et al., 2016). Recently, the relationship between GCase activity and α-syn levels has been investigated in the blood of PD and GD patients, highlighting an inverse relationship between GCase activity and plasma oligomeric α-syn levels in subjects carrying GBA1 mutations (Nuzhnyi et al., 2015; Pchelina et al., 2017). However, GCase appears to play a more general role in the pathogenesis of PD, as suggested by the decreased GCase activity detected in brain samples with increased α-syn levels and in CSF from sporadic PD patients without GBA1 mutations (Murphy and Halliday, 2014; Chiasserini et al., 2015; Rocha et al., 2015; Parnetti et al., 2017). Nevertheless, there is a substantial lack of information on GCase changes and their association with α-syn modifications in the blood of PD patients in absence of GBA1 mutations.

The general objective of this exploratory study was to identify peripheral changes potentially reflecting PD pathological status in a cohort of 39 patients with sporadic PD, without GBA1 mutations, and 33 age-matched controls, focusing the attention on the relationship between GCase activity and plasma α-syn levels. To this aim we first evaluated plasma α-syn content, associated or not with exosomes, in order to: (1) determine if PD patients exhibit altered levels of plasma α-syn; and (2) investigate whether changes in α-syn content could reflect the course of the disease and its severity. Exosome associated-α-syn content was evaluated in the total pool of plasma exosomes, taking into account the multisystemic nature of PD. Moreover, we measured GCase activity in lymphocytes and investigated whether a potential relationship with plasma α-syn levels exists.

## Materials and Methods

### Subjects and Sample Collection

PD patients without heterozygous GBA1 mutations were recruited at the C. Mondino National Neurological Institute. All patients underwent genetic testing to exclude the possibility of major GBA1 mutations (N370S, L444P, E326K polymorphism). Age-matched healthy controls were enrolled both at the C. Mondino and at IRCCS Policlinico S. Matteo Foundation. Subjects were submitted to a complete examination at enrolment. Patients and controls with a history of autoimmune or inflammatory disorders and/or receiving chronic immunosuppressive treatment were excluded. PD patients were staged according to the criteria of Hoehn and Yahr (H&Y) and evaluated by the Unified Parkinson’s Disease Rating Scale (UPDRS) Part III. Demographic and clinical data of PD patients and healthy donors are summarized in Table 1. This study was carried out in accordance with the recommendations of “IRCCS San Raffaele Scientific Institute committee” (register number: 35/int/2016) with written informed consent from all subjects. All subjects gave written informed consent in accordance with the Declaration of Helsinki. The protocol was approved by the “IRCCS San Raffaele Scientific Institute committee.” All subjects underwent venous blood sampling (35 mL) after overnight fasting.

**Table 1:** Clinical and demographic data of subjects enrolled for the study.

	Controls	PD patients	*p*-value
*n* (gender)	*n* = 33 (15 M, 18 F)	*n* = 39 (25 M, 14 F)	0.11
Age (range)	61.9 ± 6.2 (47–78)	65.2 ± 8.9 (44–78)	0.10
Age at onset (years)	_	54.9 ± 9.0	
Disease duration (years)	_	9.5 ± 6.2	
Hoehn and Yahr scale	_	2.1 ± 0.7	
UPDRS scale III	_	18.9 ± 10.6	

*Results are expressed as mean ± SD. Note: statistical comparisons were performed between the study groups: Chi-Square Test and Student-*t*-test were used for gender and age comparison, respectively. The significance level assumed was 0.05*.

### Blood Specimen Processing

Blood was collected in tubes containing EDTA to prevent clotting and the separation of plasma was achieved within 2 h of blood collection. Platelet poor plasma was prepared by centrifugation for 20 min at 1000 *g* to remove blood cells followed by a centrifugation at 1600 *g* for 20 min to eliminate platelets and cell debris. Red blood cell rupture was prevented by avoiding blood sample exposure to excessive heat or cold. Hemolysis was assessed by free hemoglobin quantification and hemolyzed specimens (free hemoglobin concentration >0.02 g/L, Thomas, 2002) were excluded from further analysis to avoid preanalytical bias. The plasma was stored at −80°C until analysis. For the preparation of the peripheral blood lymphocytes (PBLs), after the plasma layer was removed, the original volume was reconstituted with phosphate-buffered saline (PBS), layered over Histopaque-1077 (Sigma), and centrifuged at 800 *g* for 20 min. After removing the upper layer, the PBL band was transferred into a different tube and PBS was added to reconstitute the original volume. PBLs were counted, using an automated cell counter (Beckman Coulter Inc.), after which the sample was split into aliquots (5 × 10^6^ cells/each) that were centrifuged at 1000 *g* for 10 min. After discarding the supernatant, PBL pellets were stored at −80°C.

### Exosome Isolation

Exosomes were isolated from plasma (1 mL) by sequential centrifugation; 0.8 μm filtered plasma was centrifuged at 20,000 *g* (Mikro 220R equipped with 1195-A rotor, Hettich) for 1 h to remove large extracellular vesicles. The supernatant was filtered through 0.2 μm filters (Pall) and ultracentrifugated at 100,000 *g* (Optima Max–XP equipped with TLA55 rotor, Beckman Coulter) for an additional hour to pellet exosomes. The pellets were processed differently depending on the type of analysis.

### Lipoprotein Evaluation

Lipoproteins were identified as a possible contaminant of extracellular vesicle preparations (Yuana et al., 2014). To exclude that lipoproteins can considerably affect exosome evaluations, levels of high-density lipoprotein (HDL) and low-density lipoprotein (LDL) were determined by Cobas *in vitro* diagnostic test system (Cobas) at the Laboratory of Clinical Biochemistry of the Institute “C. Mondino”. This test has a lower limit of detection of 0.1 mmol/L (3.83 mg/dL) for total cholesterol and 0.08 mmol/L (3 mg/dL) for HDL.

### Albumin Determination

Albumin concentration was determined in plasma and exosome pellet with a Bromocresol Green Albumin Assay (BCG; Sigma Aldrich). The detection range of albumin was between 0.1 g/dL and 5 g/dL. Exosomes are isolated as previously described (see “Exosome Isolation” section) and the pellets were resuspended in sterile-filtered 100 μL PBS (Carlo Erba). Samples were tested in duplicate. The optical density at 620 nm was determined using a microplate reader (Biotek).

### Western Blot Analysis

Exosome pellets were resuspended in ice-cold lysis buffer (Sigma) containing diluted phosphatase and protease inhibitors (Roche). After centrifugation, the supernatant was collected and protein concentration was measured using a Bicinchoninic Acid Protein Assay (Sigma). Protein lysates (30 μg) were run on 4%–12% gels using dithiothreitol or beta-mercaptoethanol as reducing agent depending on the antibody, and transferred onto nitrocellulose membranes (Biorad).

Membranes were blocked (Odyssey blocking buffer, LiCor) and incubated overnight with the primary antibodies against the exosomal marker TSG101 (1:2000, Abcam) or Alix (1:500, Novus Biologicals). As secondary antibodies, IRDye^®^ 700 (1:10,000) or IRDye^®^ 800 (1:5000) were used. Image analysis of western blots was performed using the fluorescent near-infrared Odyssey^®^ scanner and software (LiCor).

### Nanoparticle Tracking Analysis

Concentration and size distribution profile of exosomes isolated by 1 mL plasma were evaluated using a NanoSight NS300 (Malvern) as described (Fernando et al., 2017; Logozzi et al., 2017). This technique uses the properties of light scattering and Brownian motion of the particles. The path of each particle, detected by a laser light source, is recorded and calculated over time to determine their velocity. NTA (3.2) software processes the video captures and allows the user to automatically track the size distribution and number of the nanoparticles. Video were recorded at camera level 16 and five 60 s videos were registered for each sample. The exosome pellets were resuspended in sterile-filtered 100 μL PBS (Carlo Erba) and diluted at least 1:100–1:1000 to achieve an uniform particle distribution and have the exosome concentration in the working range for the NanoSight NS300.

### Transmission Electron Microscopy (TEM)

Exosome pellets were resuspended in sterile-filtered 100 μL PBS (Carlo Erba) and a 5 μl drop of this suspension was absorbed on glow-discharge 300 mesh formvar/carbon copper grids; after 2 min grids were negatively stained with 2% uranyl acetate. Samples were imaged on an EFTEM Leo912 ab (Zeiss) transmission electron microscope operating at 100 kV and digital images were recorded by a Proscan 1K slowscan CCD by iTEM Software (Olympus).

### Alpha-Synuclein Quantification

Alpha-syn concentrations in plasma exosomes and in whole plasma were assessed by using the same sandwich ELISA assay (Anaspec), according to manufacturer’s instructions, with slight modifications due to the different nature of the samples. Twenty microgram of exosome lysates and plasma (diluted 1:50 in sample dilution buffer) were added in duplicate to the wells. The optical density at 450 nm was determined using a microplate reader (Biotek). Each ELISA test included PD and control samples. One reference exosome lysate and plasma sample were added into each well to help eliminate well variations. This assay has an average lower limit of detection of 5 pg/mL of alpha-synuclein and the coefficient of variation (CV) in human body fluids was 5.03%.

### Glucocerebrosidase (GCase) Activity Assay

The enzymatic activities of GCase in peripheral blood lymphocytes was determined with the synthetic fluorogenic substrate 4-methyl umbelliferyl-b-D-glucopyranoside (4-MUG), according to commonly used methods (Papagiannakis et al., 2015; Kim et al., 2016). PBL pellets (5 × 10^6^ cells/each) were lysed for 30 min on ice in CellLytic lysis buffer (Sigma). A reaction mix was prepared (0.15 M citrate/phosphate buffer, pH 5.9; sodium taurocholate and 1 nM 4-MUG) and added to each sample and blank well. Samples were incubated 3 h at 37°C on a shaker. The standard 1 nM 4-Methylumbelliferone (Sigma) was added before reading and the reaction was stopped with 1 M Glycine, pH 10.4. Each sample was measured independently six-fold. Fluorescence (excitation: 355 nm; emission: 460 nm) was measured using a microplate reader (Molecular Devices) and the final result is reported as nmol of substrate per hour per microgram protein.

### Statistical Analysis

Comparisons between groups were carried out using Student’s *t*-test or Mann-Whitney U test. Chi-Square Test and Student-*t*-test were used for gender and age comparison, respectively. Correlations between α-syn and clinical parameters were investigated with linear regression. The relationships between the plasma α-syn levels and GCase activity were analyzed with bivariate correlation using Pearson’s correlation coefficients. All analyses have been corrected for confounding factors (age, gender and disease duration). A dedicated software (Prism 3 software; Graph-Pad Software) was used. The minimum level of statistical significance was set at *p* < 0.05.

## Results

### Characterization of Exosomes Recovered From Human Plasma

**Figure 1:**
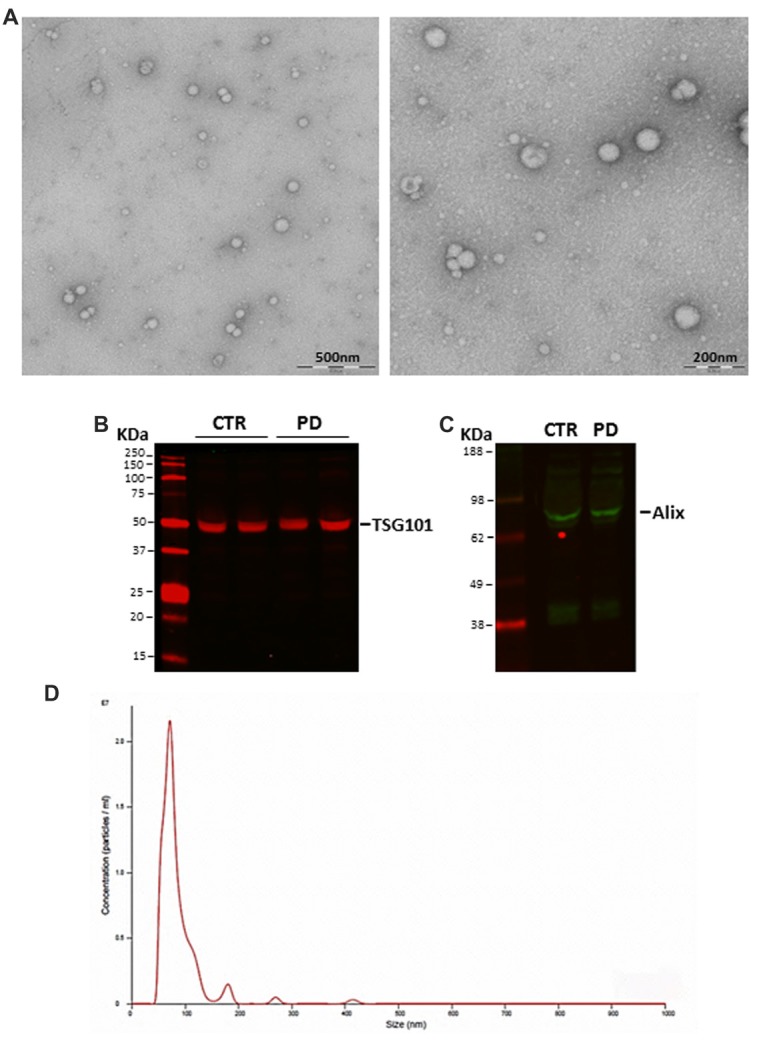
**(A)** Representative transmission electron microscopy (TEM) images of isolated exosomes. Scale bar: 200 and 500 nm. **(B)** TSG101 and **(C)** Alix expression in blood-derived exosomes as evaluated with Western blot. **(D)** Size distribution and concentration of isolated exosomes evaluated by NanoSight (CTR, controls; PD, Parkinson’s disease patients).

Exosomes isolated from plasma of healthy subjects and patients were evaluated by TEM, Western blot and Nanosight to confirm the reliability of isolation protocol and the purity of exosome preparations. The ultracentrifugation pellet contained 40–120 nm membrane-bound vesicle (Figure 1A), which displayed the exosomal markers TSG101 (Figure 1B) and Alix (Figure 1C). No differences were found between PD patients and healthy subjects in the expression of exosomal markers. Nanoparticle tracking analysis showed a particle size distribution ranging from 50 nm to 300 nm in diameter, with the most particles detected in the size range corresponding to exosome dimensions (85.8 ± 6.8 nm; mean ± SD; Figure 1D). It is noteworthy that blood plasma contains particles such as lipoproteins (predominantly LDL) that mimic the characteristics of extracellular vesicles and could also be co-purified with them. In order to evaluate whether the presence of lipoproteins in our samples could cause false positive hits in the analyses, we measured the concentration of HDL and LDL in plasma, exosome-enriched fraction and exosome-poor plasma (obtained by removing exosomes after ultracentrifugation) from independent samples. No detectable levels of lipoproteins were found in the exosome-enriched fraction, whereas total and exosome-poor plasma showed comparable concentrations of HDL and LDL (data not shown). This proves that lipoproteins did not co-precipitate with exosomes after centrifugation, in our samples. Lastly, we assessed albumin levels in our exosomal preparations since it is known that exosomes isolated using the ultracentrifuge method can be contaminated with this protein. A negligible fraction (about 5%) of plasma albumin precipitates in exosomal preparations without significant differences between control- and patient-derived samples (Supplementary Figure S1).

### Plasma Exosomal α-syn Significantly Increases in Patients With PD

Since the commercial ELISA assay kit we used was adapted for use with exosomal samples, we firstly tested the robustness of this assay. Exosomes were prepared from different aliquots of the same sample and α-syn protein levels were measured in duplicates, repeating the test four times. The intra-assay CV was below 10% and the inter-assay CV was 16.6% which is considered acceptable (Guidance for industry-bioanalytical method validation, Food and Drug Administration, 2001) corroborating the reliability both of ELISA assay and exosome preparation.

**Figure 2:**
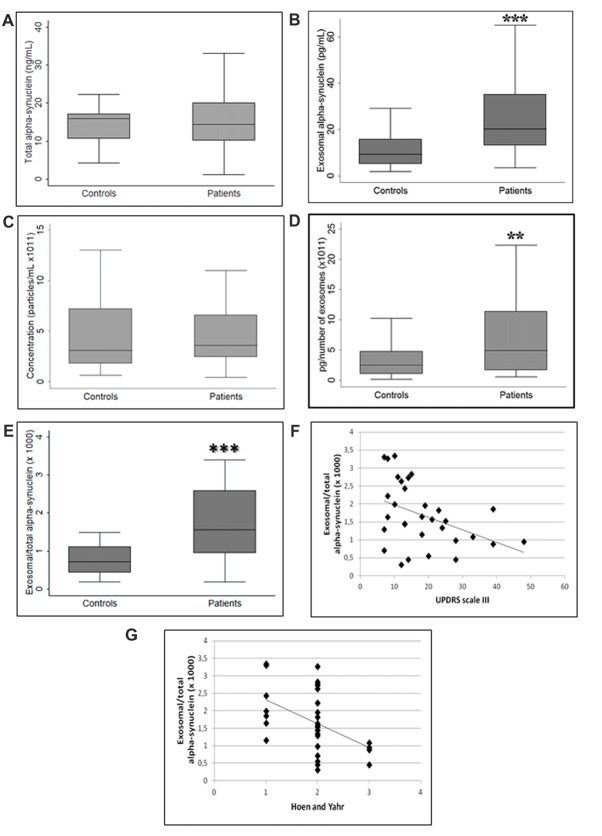
Comparison between **(A)** total alpha-synuclein (α-syn), **(B)** exosomal α-syn, **(C)** exosome concentration, ratio of exosome-associated α-syn to **(D)** exosome concentration and **(E)** total α-syn in controls and PD patients. **(F,G)** Exosomal/total α-syn ratio and disease severity correlation. **p < 0.01, ***p < 0.001 Student’s *t*-test.

No differences were found in the plasma levels of total fraction of α-syn between patients and controls (*p* = 0.53, Student’s *t*-test; Figure 2A). It should be highlighted that the term “total α-syn” essentially refers to non-exosome-associated plasma α-syn, since exosomal α-syn contributes only a paltry portion to the total (Ejlerskov et al., 2013). Conversely, α-syn concentration in plasma exosomes was higher in patients with PD compared to healthy controls (*p* < 0.001, Student’s *t*-test; Figure 2B). We then quantified the concentration of plasma exosomes in order to verify whether exosomal α-syn increase was indeed attributable to the increment of exosome protein content or vesicle concentration. As depicted in Figure 2C, analysis of exosome concentration in plasma showed higher variability among healthy subjects compared with patients, without significant differences between the groups (*p* = 0.76, Mann-Whitney U test). However, the exosomal α-syn to particle concentration ratio was significantly augmented in PD patients, confirming that changes in exosome-associated levels of α-syn were correlated with the pathological status (*p* < 0.01, Mann-Whitney U test; Figure 2D). Similarly, the plasma exosomal α-syn/total α-syn ratio increased significantly (*p* < 0.001, Student’s *t*-test) in PD subjects as compared to controls (Figure 2E). Correlation analysis revealed a significant negative correlation between this ratio and the measures of disease severity, indexed by the UPDRS part III (*r* = −0.4174, *p* = 0.019; Figure 2F) and H&Y (*r* = −0.4608, *p* = 0.009; Figure 2G) scores in PD patients. However, the relationship between plasma exosomal α-syn/total α-syn ratio and UPDRS score was found to be insignificant (*p* = 0.10) after confounders (age, gender and disease duration) were taken into account whereas we found that this ratio negatively correlated with H&Y score (*p* = 0.014), even after considering confounder effects.

### GCase Activity Negative Correlates With Exosome-Associated α-syn

**Figure 3:**
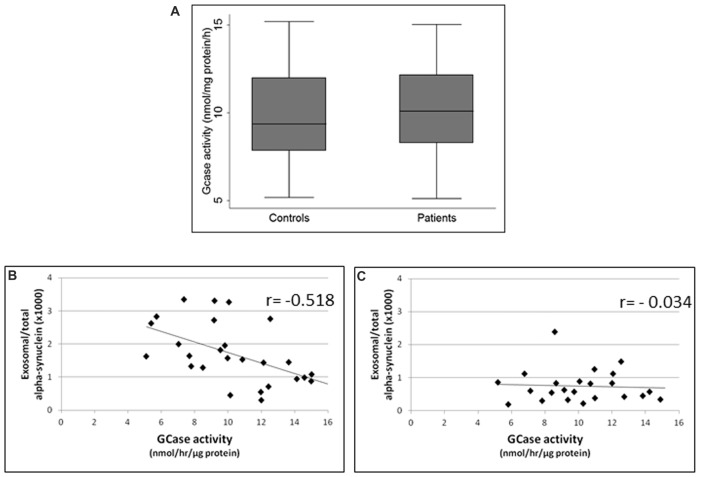
**(A)** Glucocerebrosidase (GCase) activity in controls and PD patients. Correlations between exosomal/total α-syn ratio and GCase activity in **(B)** PD patients and **(C)** controls.

GCase enzymatic activity in lymphocytes did not differ between PD patients and healthy subjects (*p* = 0.5, Student’s *t*-test; Figure 3A). However, it is noteworthy that GCase activity was inversely correlated with exosomal α-syn/total α-syn ratio in PD subjects (*r* = −0.518, *p* = 0.006; Figure 3B), whereas no significant correlation was found between these parameters in controls (*r* = −0.034, *p* = 0.878; Figure 3C).

## Discussion

There is a growing need for more specific and cost-effective biomarkers in neurodegenerative diseases that can work in conjunction with clinical assessment and imaging to diagnose patients at earlier stages and more accurately, as well as to predict disease outcome, or the response to a given therapy. The discovery of α-syn as a major component of Lewy bodies suggested the investigation of this protein as a biomarker.

In this study, we investigated whether changes in plasma total and exosome-associated α-syn could correlate with disease status and clinical parameters in PD and their relationship with GCase activity, since mutations in this α-syn-interacting enzyme are the most important risk factor for PD.

Blood appears to be the most suitable source for biomarker discovery, offering easy access and handling. Moreover, alterations in the blood compartment could intrinsically reflect a more widespread disease showing both central and peripheral manifestations. However, despite the large number of published studies, the findings on α-syn in blood as PD biomarker have been inconsistent to date. Conflicting results may be mainly attributable to biological confounders, such as red blood cell contamination during sample collection, these cells being the major source of α-syn in blood (Barbour et al., 2008), as well as sample processing and variations in standardization of methods (Mollenhauer et al., 2017). These factors can also potentially affect the evaluation of exosome-associated α-syn, another intriguing source for α-syn in blood that is still relatively unexplored. In order to overcome these concerns, we optimized our sample processing by taking into account technical suggestions from the International Society for Extracellular Vesicles (ISEV; Witwer et al., 2013) and a more recent article on α-syn assessment in biological fluids (Mollenhauer et al., 2017). Potential blood contamination of plasma samples has been estimated by measuring hemoglobin concentration and hemolyzed samples were excluded to avoid inaccurate plasma α-syn measurement due to erythrocytes-derived α-syn adulteration. The reliability of exosomal isolation protocol and the purity of exosomal preparations have been verified by TEM, Western blot and NTA analyses. Moreover, the absence of relevant plasma lipoprotein concentration in our exosomal preparations can ruled out that exosome concentration measurement have been invalidated. The most challenging issue in our protocol could be albumin contamination. Albumin has been found in our samples—though in small amounts and at comparable levels between control- and patient-derived samples—suggesting that it could potentially impact the exosomal content evaluation in a similar way on both groups. Although the insertion of an additional washing step in the isolation protocol can further reduce albumin contamination, this is accompanied by a noticeable loss of exosomes that can cause the loss of informative data. Therefore, bearing this issue in mind, we believe that the proposed method can be used reliably for the evaluation of the protein content, such as α-syn, in exosomes.

Our results showed that exosomal α-syn content was significantly higher in PD patients as compared to controls. This difference was not affected by individual exosome concentration, confirming that blood-derived exosomes from PD patients contain a greater amount of α-syn per exosome compared to healthy subjects. Conversely, no differences were found in plasma total α-syn levels between patients and healthy subjects. The significant increase of exosome-associated α-syn in PD patients could reflect the functional role played by this protein “source” in PD pathology. Danzer et al. (2012) showed that exosome associated α-syn was more toxic to cells than the exosome-free protein corroborating that this pathway of protein release could be underlying the spread of α-syn pathology observed in human post-mortem brains. Accordingly, several findings suggested that exosomal α-syn species from patients with α-synuclein related neurodegeneration serve as carriers for interneuronal disease transmission, by inducing oligomerization and aggregation of the endogenous protein (Stuendl et al., 2016; Ngolab et al., 2017). Despite the lack of difference between patients and controls in plasma total α-syn levels is in keeping with the findings of Shi et al. (2014), they observed an increase of exosomal α-syn content in PD patients entirely deriving from exosomes of neuronal origin (i.e., L1CAM-positive). Although the α-syn increment we observed in the total pool of plasma exosomes may seem less specific, it could indeed reflect the systemic nature of PD. Exosomes can be secreted by virtually all cell types, including peripheral blood cells (Zitvogel et al., 1998; Matsumoto et al., 2017), and several studies indicate that α-syn levels are increased in red blood cells and peripheral lymphocytes of PD patients (Kim et al., 2004; Wang et al., 2015; Matsumoto et al., 2017). Thus, we can hypothesize that different cell populations contribute to the increased plasma exosomal α-syn in PD patients, generally recapitulating systemic changes involved in PD status. Moreover, since our protocol does not require critical steps for exosomal subpopulation isolation and allows to obtain a sufficient amount of vesicles from small sample volume, the evaluation of α-syn levels in the total exosomal pool may be practical for use in a routine clinical setting after further optimization.

We also highlighted a significant increase of plasma exosomal α-syn/free α-syn ratio in PD patients, which negatively correlated with disease severity indexed by the H-Y score even after considering the confounder effects. This negative relationship, suggesting an increase of non-exosome-associated α-syn fraction vs. exosome-associated one with disease severity, could be attributable to a change in the equilibrium between these forms of the protein. The preferential mechanism through which α-syn is released from cells is still matter of study. As mentioned above, exosomes—which originate from the fusion of multivesicular bodies (MVB) with the plasma membrane—seem to preferentially transfer pathological species of α-syn. Moreover, Hasegawa et al. (2011) have demonstrated that under physiological conditions, α-syn is destined for lysosomal degradation or released into the extracellular milieu as free fraction and, to a lesser degree, in association with exosomes. However, if the intracellular α-syn reaches a toxic level or MVB sorting is impaired by α-syn accumulation (Schreij et al., 2016), excessive amounts of α-syn flow out mainly as free form. Thus, we can hypothesize that increasing accumulation of α-syn during disease progression can potentially account for the displacement of plasma exosomal α-syn/free α-syn ratio toward non exosome-associated α-syn.

In parallel with α-syn, we evaluated the activity of lysosomal enzyme GCase in lymphocytes. Peripheral GCase activity was unchanged between PD patients and healthy subjects, in keeping with results obtained from previous studies conducted in PD patients without GBA1 mutations (Papagiannakis et al., 2015; Kim et al., 2016). One of the most intriguing results of this study was the strong inverse correlation between GCase activity and exosomal α-syn/total α-syn ratio in PD subjects, suggesting that changes in GCase activity may be involved in process regulating α-syn release through exosomes. Recent studies have tried to examine whether a relationship between α-syn and GCase in peripheral body fluids exists (Nuzhnyi et al., 2015; Pchelina et al., 2017). However, to our knowledge this is the first study correlating GCase activity and exosomal α-syn in blood. Few studies so far have examined the effect of GCase activity on extracellular α-syn release. Bae et al. (2014) demonstrated that GCase deficiency leads to increased release of aggregated α-syn into the extracellular space, and more cell-to-cell transfer and seeding of α-syn aggregates in the host. Accordingly, GBA-N370S dopaminergic neuronal cultures showed a notable release of α-syn in the culture media (Fernandes et al., 2016). In addition, systemic administration of the lysosomal inhibitor Bafilomycin A1 induced a marked increase of α-syn levels in the CSF of wild-type and α-synuclein transgenic mice (Poehler et al., 2014). Most interestingly, *in vitro* experiments showed that lysosomal dysfunction led to an increase in the release of α-syn in exosomes and a concomitant increase in α-syn transmission to recipient cells (Alvarez-Erviti et al., 2011) corroborating our results.

In conclusion, our results highlight peripheral changes suggestive of PD pathological status and indicate that association between plasma exosomal α-syn/total α-syn ratio and PD is complex, as it may reflect the presence, as well as the stage of the disease. The inverse correlation between peripheral GCase activity and exosome-associated α-syn suggests a potential mechanism by which changes in GCase activity may affect the exosome/total α-syn balance, thereby contributing to PD pathogenesis. Additional and large-scale studies comparing GCase activity and pathological protein levels will be clearly needed to corroborate these data and determine whether the association between key players in the lysosomal system and α-syn can be used as diagnostic or prognostic biomarkers for PD.

## Author Contributions

SC and FB: study concept and design. SC, CG, MS, FS, RZ, BM, MA and GD: acquisition and analysis of the data. SC and FB: drafting the manuscript and/or figures.

## Conflict of Interest Statement

The authors declare that the research was conducted in the absence of any commercial or financial relationships that could be construed as a potential conflict of interest. The reviewer AM and the handling editor declared their shared affiliation.
