# Supplementary figures and images for: The Exosomal/Total α-Synuclein Ratio in Plasma Is Associated With Glucocerebrosidase Activity and Correlates With Measures of Disease Severity in PD Patients
Source: Front Cell Neurosci. 2018 May 18;12:125. doi: 10.3389/fncel.2018.00125 (PMC5968118; doi:10.3389/fncel.2018.00125)

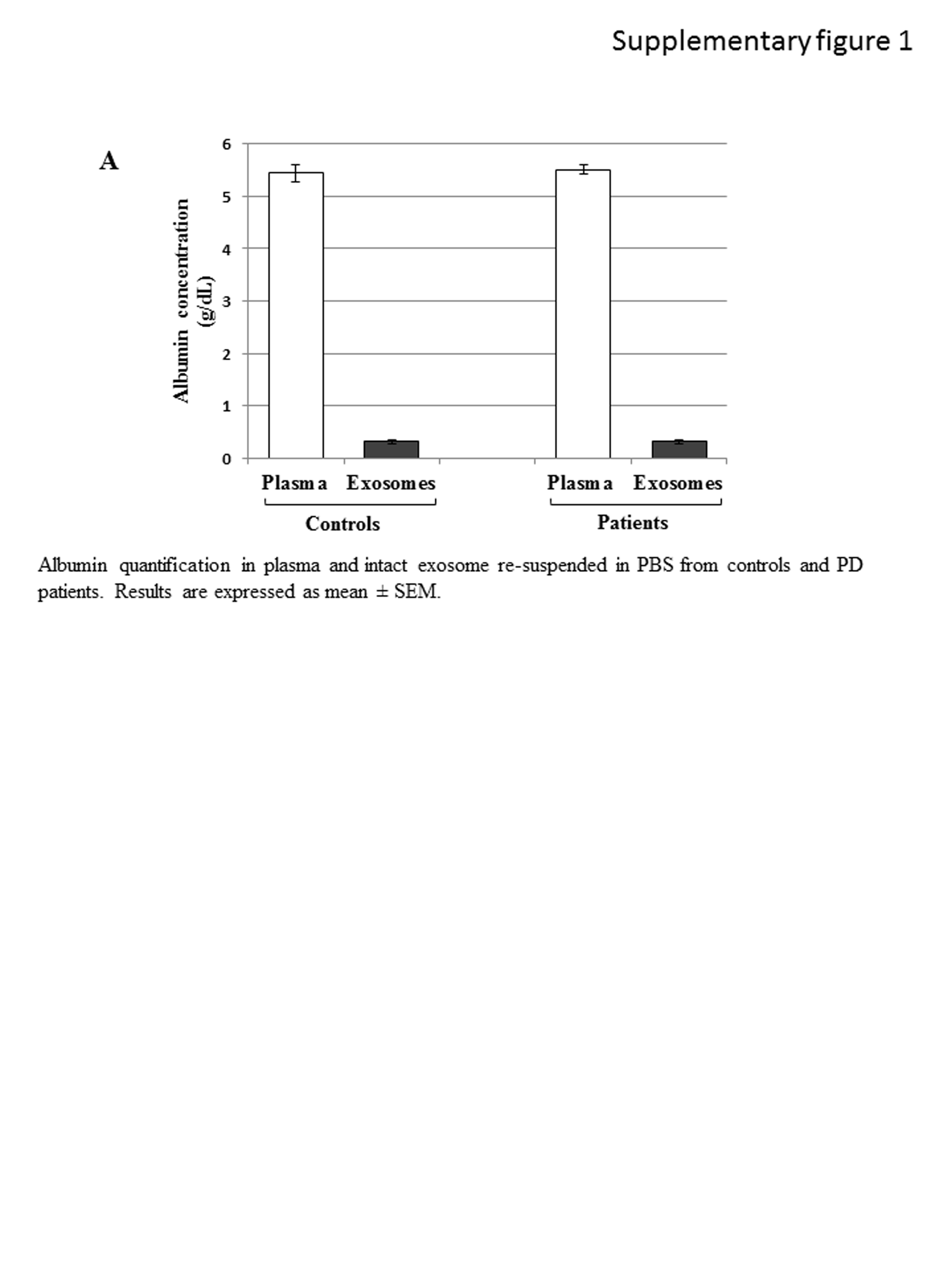

Supplement: Supplementary file 1 [file Image_1.tif]
